# Improving Statin Prescribing for People Living With HIV: A Quality Improvement Initiative

**DOI:** 10.1016/j.jaccas.2026.109018

**Published:** 2026-06-27

**Authors:** Veronica Njuguna, John Hawkins, Navya Pothamsetty, Daniel Chen, Alyson Brinkman, Nirmala D. Ramalingam, Christian Lee-Rodriguez, Mitchell N. Luu

**Affiliations:** a Graduate Medical Education, Kaiser Permanente Oakland Medical Center, Oakland, California, USA; b Kaiser Permanente Northern California Division of Research, Pleasanton, California, USA; c Department of Infectious Diseases, Kaiser Permanente Oakland Medical Center, Oakland, California, USA; d Department of Internal Medicine, Kaiser Permanente Northwest, Portland, Oregon, USA

**Keywords:** ASCVD, HIV, quality improvement, statin

## Abstract

**Background:**

People living with HIV (PLWH) have increased atherosclerotic cardiovascular disease risk. Guidelines recommend statin therapy for PLWH aged 40 to 75 years with a 10-year atherosclerotic cardiovascular disease risk of ≥5%.

**Project Rationale:**

Statin prescribing is suboptimal in this population. We implemented a quality improvement initiative to identify barriers and increase statin prescribing in eligible PLWH.

**Project Summary:**

Seventeen of 25 (68%) Kaiser Permanente HIV specialists were surveyed on barriers to statin prescribing and participated in a quality improvement initiative that provided monthly education and prescribing feedback. Statin prescriptions among PLWH aged 40 to 75 years were assessed before the intervention (June-December 2024) and during the intervention (January-June 2025) using interrupted time series. Seventeen of 25 specialists (68%) participated. Reported barriers included patient reluctance (71%), nonautomated risk calculation (58%), and limited resources (47%). Baseline statin prescribing was 44.1%. Monthly prescribing increased from 0.30% preintervention to 1.24% postintervention (*P* < 0.001), yielding 193 additional prescriptions.

**Take-Home Message:**

An intervention combining ongoing education with monthly performance feedback significantly improved statin prescribing rates among PLWH.

## Visual Summary

**Figure:**
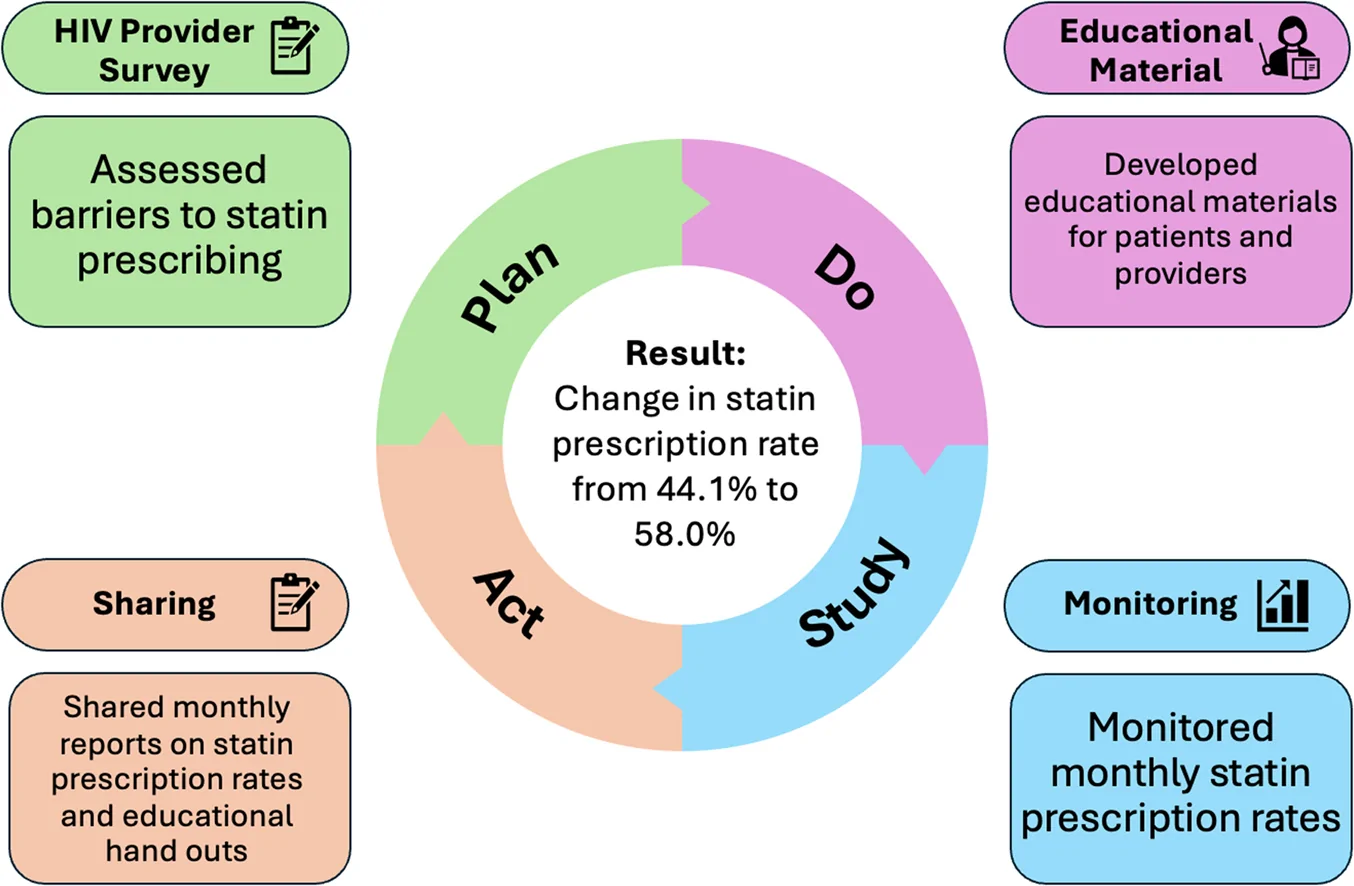

People living with HIV (PLWH) have an increased risk of atherosclerotic cardiovascular disease (ASCVD) attributed to a combination of chronic immune activation and inflammation associated with HIV infection, as well as antiretroviral therapy–related metabolic disturbances, including dyslipidemia.^1^ Despite having a significantly elevated risk for ASCVD, PLWH have low statin prescribing rates.^2^

The REPRIEVE (Randomized Trial to Prevent Vascular Events in HIV) trial demonstrated a 35% reduction in major adverse cardiovascular events with pitavastatin among PLWH aged 40 to 75 years with a 10-year ASCVD risk of >5%.^3^ With these data, the U.S. Department of Health and Human Services, in collaboration with the American College of Cardiology, American Heart Association, and the HIV Medicine Association, updated guidelines in February 2024 and recommended at least moderate-intensity statin therapy for PLWH aged 40 to 75 years with a 10-year ASCVD risk of 5% to 20%.^4^

Our goal was to conduct a quality improvement (QI) project to identify barriers, provide education, and increase statin prescribing among HIV specialists at Kaiser Permanente (KP) East Bay and San Francisco in accordance with these guidelines.

## Project Rationale

Despite strong evidence and updated guidelines, statin prescribing among PLWH remains suboptimal. Less than 50% of PLWH at risk for ASCVD have been prescribed statins at KP. We aimed to identify provider barriers to statin prescribing and implement interventions to improve guideline-concordant prescribing.

## Project Description

### Setting and population

This QI project occurred during June 2024 to June 2025, with retrospective collection of baseline data for the period before the QI project (June 2024 to December 2024) and the period during the QI project intervention (January 2025 to June 2025), at 2 KP Northern California medical centers: KP East Bay and KP San Francisco. KP Northern California is a large integrated health care system with approximately 1,400 and 2,800 PLWH receiving care at KP East Bay and KP San Francisco, respectively.

### Survey and educational intervention

A preintervention survey (Supplemental Appendix A1) was developed in Microsoft Forms and sent to HIV specialists, which included internal medicine, family medicine, and infectious disease physicians, at KP East Bay and KP San Francisco to determine familiarity with updated statin guidelines, assess current prescribing practices, and identify barriers to prescribing. In January 2025, the survey was distributed to 25 HIV specialists at both sites via 3 separate emails. Participation was voluntary.

After survey completion, the HIV specialists received a brief educational handout summarizing new guidelines, recommended statins and dosages based on antiretroviral therapy regimen, and patient education resources developed by the authors (Supplemental Appendix A2 and A3).

### Data collection

Data on PLWH aged 40 to 75 years who received care at KP East Bay or KP San Francisco between June 2024 and June 2025 were gathered from the KP Northern California HIV Registry. Variables collected included age, 10-year ASCVD risk score and calculation date, and statin prescription details (date, drug, and dose).

### Statin prescribing reports

On January 2025, monthly prescribing reports (Supplemental Appendix A4) were distributed to participating HIV specialists. Reports included the number of eligible patients, the number and percentage prescribed statins, and prescribing rates by age group (40-49, 50-59, 60-69, 70-75 years). Site-level comparisons between East Bay and San Francisco were also provided.

### Statistical analysis

Interrupted time series analysis was used to evaluate the impact of the intervention, implemented on January 1, 2025. The primary outcome was the monthly percentage of eligible patients prescribed statins.

Segmented linear regression models estimated prescribing trends before and after the intervention for East Bay, San Francisco, and the combined cohort. The preintervention slope represented the monthly change in statin prescribing before the intervention, the immediate change represented the level change in prescribing at the time of intervention relative to the projected preintervention trend, and the postintervention slope represented the monthly change in prescribing after the intervention.

## Project Deliverables

1. Preintervention clinician survey identifying prescribing barriers
2. Educational materials summarizing statin guidelines and patient resources
3. Monthly prescribing reports with site-level performance comparisons.

## Project Outcome

Seventeen of 25 HIV specialists (68%) participated in the QI program (8 East Bay and 9 San Francisco). Of these specialists, 3 (17.6%) were somewhat familiar, 10 (58.8%) were familiar, and 4 (23.5%) were very familiar with the updated 2024 US Department of Health and Human Services guidelines. All HIV specialists felt comfortable prescribing statins, with 76% reporting feeling very comfortable. When assessing ASCVD risk, 96% reported that they routinely screen based on risk factors. Prescribing barriers included negative reactions from patients (71%), lack of automated ASCVD score (58%), lack of patient resources (47%), low priority at the time of visit (41%), fear of drug-drug interaction (24%), and patients’ lack of awareness about the new guidelines (24%).

For both medical centers, the baseline rate of statin prescription for eligible members at the start of the observation period was 44.08%. Before the intervention, statin prescribing was increasing at a baseline of 0.30% per month (95% CI: 0.25-0.34). There was no significant change immediately after the intervention (0.34%, 95% CI: −0.78 to 1.47). However, the postintervention slope increased significantly to 1.24% per month (95% CI: 0.95-1.53). Compared with the counterfactual scenario in which no intervention occurred, we estimated that 193 additional patients were prescribed statins for the duration of the QI project.

**Figure 1:**
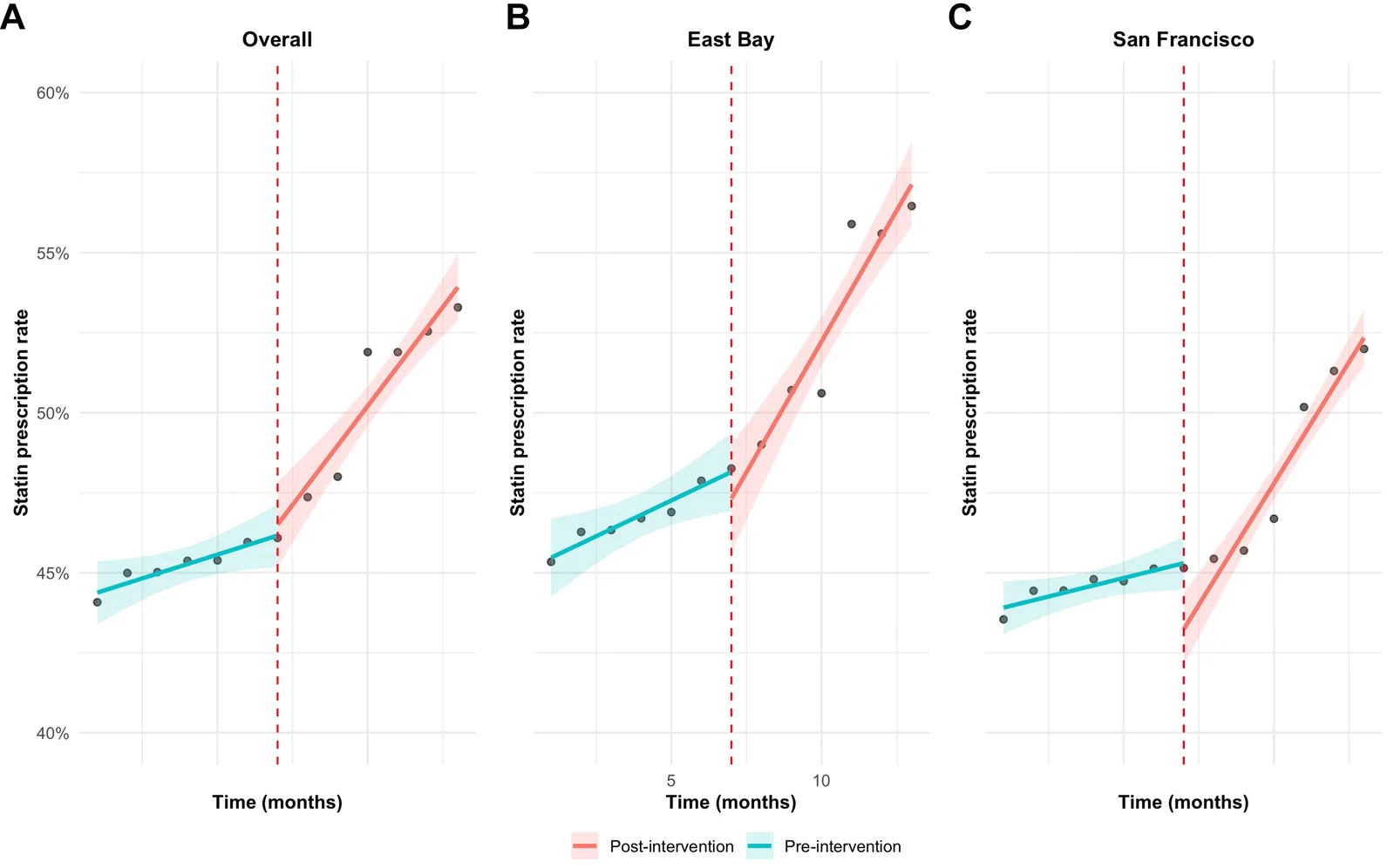
Interrupted Time Series of Statin Prescription Rates Before and After Quality Improvement Intervention In these graphs, the points show observed monthly statin prescription rates; the solid lines indicate model-predicted rates with 95% confidence intervals (shaded). The dashed red line indicates the intervention. Trends are shown overall (A) and by site: East Bay (B) and San Francisco (C).

Medical center analyses showed similar patterns. Regression results for the overall cohort and both sites are depicted in Figure 1. A complete summary of regression results is provided in Supplemental Appendix B.

## Impact and Future Directions

Our QI project significantly enhanced statin prescribing for PLWH by HIV specialists at KP East Bay and San Francisco. Future efforts will focus on improving patient education, addressing workflow barriers, and expanding interventions at additional sites.

## Discussion

Our QI project highlighted that statin therapy was underused among PLWH at KP and that targeted interventions improved prescribing. Overall, statin prescribing increased by 9.21% from June 2024 to June 2025.

Our survey data identified negative patient perceptions as the most frequently reported barrier, even among clinicians who were otherwise comfortable prescribing statins. This finding underscores the importance of providing tools to educate and address misconceptions about statin therapy. In response, our intervention intentionally incorporated patient-facing educational materials and handouts designed to address common misconceptions about statin safety and necessity. A 2022 meta-analysis strongly supports this work, showing no significant association with myalgia, elevated liver enzymes, or creatine kinase.^5^ In addition, a qualitative study in 2024 suggests that PLWH are more willing to take statins when educated by their provider.^6^ Taken together, our findings suggest that provider-directed education and tools, such as the patient education handouts used in our QI project, are essential to improving guideline-concordant prescribing. Equipping clinicians with targeted educational tools may be a key mechanism driving the observed increase in statin use.

Another barrier identified among our HIV specialists was the lack of automated ASCVD risk calculators. Our project addressed this barrier by including guidance in the physician educational material on how to access automated ASCVD risk calculators in the electronic health record (EHR). Literature evaluating whether this approach improves prescribing practices is limited. However, one recent small-scale study demonstrated the use of EHR-integrated ASCVD risk scores to be substantially associated with an increase in statin prescribing.^7^ Improved access to automated ASCVD risk estimators is a promising approach to increase guideline-recommended statin prescribing for PLWH.

Our project also highlights the value of reiterative feedback through monthly statin prescribing reports. These reports provide data and likely influenced clinician behavior in several different ways. First, regular feedback increases awareness of one’s own practice patterns, particularly in clinical settings where statin prescription may be deprioritized. Second, the inclusion of site-level comparisons introduces a benchmarking effect, allowing clinicians to compare their performance with peers and motivating improvement. Finally, the longitudinal nature of monthly reporting reinforces accountability over time. This approach aligns with prior literature demonstrating that audit-and-feedback interventions can meaningfully improve clinician performance.^8^ Our QI project used this approach and found a 9.21% increase in statin prescribing.

There were several limitations to our project. Despite multiple outreach emails, only 17 of 25 HIV specialists at 2 sites participated, which may impact the generalizability of the intervention. In addition, KP Northern California uses a validated HIV registry with integrated EHR, including laboratory and pharmacy data, which may limit reproducibility from nonintegrated health systems.

**Visual Summary:**
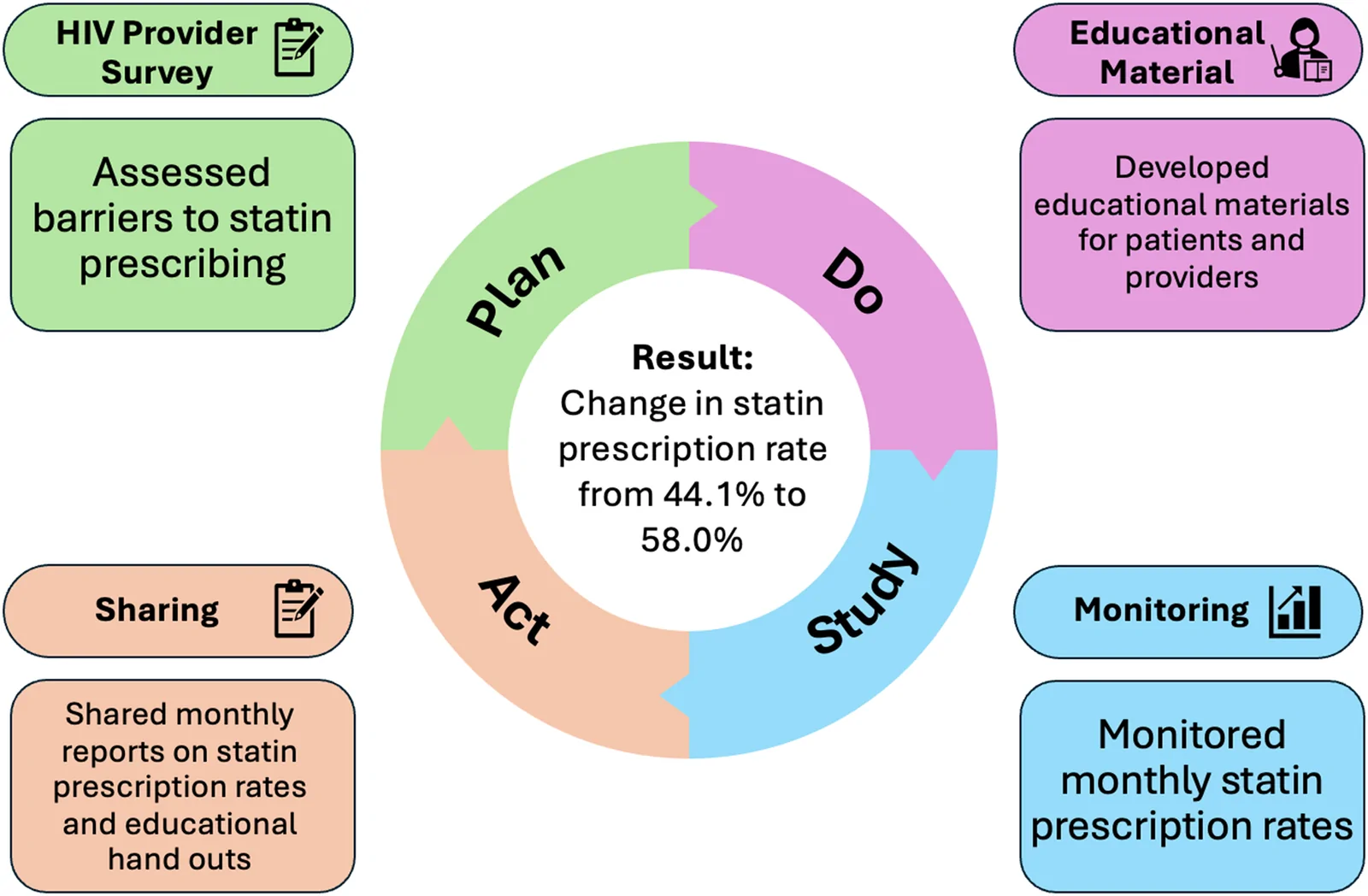
Enhancing Statin Prescribing Practices This visual summary shows how our facility concordant quality improvement intervention of provider and patient education paired with monthly feedback was associated with significant improvement in statin prescribing rates among HIV providers for people living with HIV.

## Conclusions

A QI project with educational materials and monthly reports was associated with a significant increase in statin prescribing among PLWH. These findings suggest that low-intensity, scalable interventions, such as clinician education, patient-facing materials, and performance feedback, can increase uptake of guideline-recommended cardiovascular prevention therapies and improve cardiovascular outcomes among PLWH.

## Funding support and author disclosures

The authors have reported that they have no relationships relevant to the contents of this paper to disclose.

**Take-Home Messages**

• Statin prescribing barriers include negative reactions from patients, lack of automated atherosclerotic cardiovascular disease score, and lack of patient resources.
• Reiterative clinician education and monthly performance feedback significantly improved prescribing rates for people living with HIV.

## Supplementary materials

Supplemental Appendices 1-4
