# Supplementary material for: Improving Statin Prescribing for People Living With HIV: A Quality Improvement Initiative
Source: JACC Case Rep. 2026 Jun 27;31(33):109018. doi: 10.1016/j.jaccas.2026.109018 (PMC13523448; doi:10.1016/j.jaccas.2026.109018)
Supplement: Supplementary file 1 [file mmc1.pdf]

Appendix A. Quality Improvement Project Materials

Appendix A1. Survey

Improving Statin Prescribing for Patients Living with HIV at Risk for ASCVD

Question 1: I am familiar with the new 2024 guidelines about prescribing statins for patients living with HIV

- Very familiar
- Familiar
- Somewhat familiar
- Not familiar

Question 2: I feel comfortable prescribing statins to patients living with HIV on my panel

- Very comfortable
- Comfortable
- Somewhat comfortable
- Not comfortable

Question 3: Barriers to prescribing statins for my patients living with HIV include (please check all that apply):

- Lack of awareness about the new guidelines
- Negative reaction from patients
- Fear drug-drug interactions
- Low priority at time of visit
- Don’t know which statins are appropriate
- Don’t know appropriate dosage

- Lack of patient education resources
- No automated ASCVD score
- Other (free response)

Question 4: Which best describes how often you check ASCVD for patients:

- I screen based on risk factors
- I routinely screen
- I do neither
- I do both

Question 5: What are some of the reasons why your patients decline statin prescriptions?

(free response)

Question 6: Please feel free to provide any resource you use when counseling your patients about statin prescriptions?

(free response)

Appendix A2. Provider Education Resource

Improving Statin Prescribing for Patients Living with HIV at Risk for ASCVD

People living with HIV (PLWH) are two times more likely to develop heart disease. Statin prescription for PLWH has traditionally been low because of lack of standardized guidelines and concerns for drug-drug interactions with HIV medications. The 2023 study REPRIEVE [click link] emphasized the importance of primary prevention statin prescription among patients with HIV by showing a significant reduction in major adverse cardiovascular events (MACE). On February 2024, after this study the was published the US Department of Health and Human Services, ACC, AHA, HIVMA to collaborated to develop following updated guidelines on statin prescriptions for PLWH.

**AGE**		**RECOMMENDATION**
**40-75**	ASCVD risk 5%-10%	Pitavastatin 4mg , **Atorvastatin 20 mg or Rosuvastatin 10 mg daily. Perform further risk assessment to consider using a high-intensity statin.
	ASCVD risk >20%	High intensity statin

**Given pitavastatin is not formulary internal expert recommendation would be to start with rosuvastatin or atorvastatin (if not on a booster)

Further guidance on **choosing statins** [click link] and dosage while on ARV

Resources for drug-drug interactions by order of preference

Liverpool HIV Interactions

Appendix A3. Patient Education Resource

HIV, Statin & Me

What is cardiovascular disease?

"Cardiovascular disease" includes both heart and blood vessel problems (including coronary disease), many of which are related to a process called atherosclerosis.

Atherosclerosis (known as “hardening of the arteries") develops when a substance called plaque builds up in the walls of the arteries.

This buildup narrows the arteries, making it harder for blood to flow through.

Sometimes a blood clot forms and can block the blood flow.

This can cause a heart attack or stroke, which can dramatically affect a person’s day to day life.

How does this affect me and what does this have to do with my HIV?

People living with HIV have higher rates of heart attack or stroke. About 50% (1 in 2) of all people living with HIV have plaque in their heart’s arteries, some without ever experiencing chest pain or other symptoms.

There is some increased risk due to well-known factors, including high cholesterol, diabetes, high blood pressure, smoking, and genetics or family history.

Research suggests that inflammation from HIV infection, even when it’s well-controlled, can increase plaque and make it more likely to cause heart attack or stroke.

A small number of HIV medications are associated with increased heart attack and stroke.

How do I prevent a heart attack or stroke from happening to me?

There are many important ways you can prevent heart attack or stroke:

Taking antiretroviral therapy (HIV meds) without breaks

Eat well and exercise daily

Keep blood pressure, blood sugar, and cholesterol in good range

Avoid smoking and stimulant drug use; limit the amount of alcohol you drink

In addition, STATIN medications are very effective, in particular for people living with HIV. These medications were initially developed to decrease LDL ("bad") cholesterol, but now we know they help *even if cholesterol is normal.*

In 2024, guidelines recommend a statin medication for people living with HIV who are at least 40 years old to reduce cardiovascular events (heart attack or stroke). This is based on a new research study "REPRIEVE," a randomized control trial of more than 7,700 patients living with HIV in multiple countries, which showed a decrease (fewer) cardiovascular events by about 35% over 5 years in patients receiving pitavastatin instead of placebo.

What are the side effects of statin medications?

Side effects are very uncommon and typically improve over time or with adjustments in the dose or type of medication.

Less than 1 in 10 people notice mild headache, gas, or bloating/abdominal discomfort.

Less than 4 in 100 people notice muscle aches or weakness

Very rarely people can experience increased blood sugar, or cause more serious muscle pain

In the REPRIEVE study, HIV+ patients taking pitavastatin had 0.21% (2 in 1000) increased rate of bothersome muscle aches or weakness compared to placebo.

My cholesterol is normal, why do I need a medication to lower it?

In people living with HIV, statins work to decrease risk of heart attack and stroke in multiple ways:

Lower LDL ("bad") cholesterol

Decrease HIV-associated inflammation

Shrink and stabilize fatty plaque in the heart's arteries

Is there a way to know my own personal risk of heart attack or stroke?

Your doctor can look at your overall health, age, cholesterol, and other factors to roughly estimate your risk.

You can see how a statin would decrease your risk by using online tools

We recommend a statin for everyone living with HIV over the age of 40, and strongly recommend a statin for anyone with a 10-year ASCVD risk over 5%.

It's hard to take more than one medication, should I take a statin instead of my HIV medication?

No. Regular, uninterrupted HIV treatment (anti-retrovirals) is the most important thing you can do to prevent long term risk of severe health problems, including heart attack and stroke.

How do I take a statin?

A statin is a tablet taken one time everyday. It can be taken with your other medications, including HIV medications.

Will I have to take a statin for the rest of my life?

We do recommend taking a statin long-term since the risk of heart attack and stroke increases as we age. If you would like to stop or change your medication, this is always possible, and you can talk to your doctor about the risks and benefits.

Which statin can I take? What about interactions with my HIV medications?

Atorvastatin ("Lipitor") and Rosuvastatin ("Crestor") are the most common statins and are the most compatible with HIV medications. Your doctor will review your specific medication, vitamin, and supplement list before you are prescribed any new medication and can adjust your dose if needed.

When should I NOT take a statin?

If there is a chance that you are pregnant, have a serious liver problem, or have had an allergic reaction to a similar medication, then a statin is not right for you.

Information above was adapted from NIH guidelines and REPRIEVE study results at:

https://clinicalinfo.hiv.gov/en/guidelines/hiv-clinical-guidelines-adult-and-adolescent-arv/statin-therapy-people-hiv

https://www.reprievetrial.org/learnmore/results-of-the-reprieve-trial/

Appendix A4. Example Monthly Report Sent by email.

Dear HIV Providers of East Bay and San Francisco,

Here is the current data as of February 2025:

	Feb-2025		Jan-2025	
	KP East Bay	KP San Francisco	KP East Bay	KP San Francisco
Total patients ages 40-75, n	xxx	xxx	xxx	xxx
Total on statin, n	xxx	xxx	xxx	xxx
Patients on statins by age group, %				
40-49	xxx%	xxx%	xxx%	xxx%
50-59	xxx%	xxx%	xxx%	xxx%
60-69	xxx%	xxx%	xxx%	xxx%
70-75	xxx%	xxx%	xxx%	xxx%

Physician resources: Review the handout attached to this email. This includes resources and information on guideline, calculating ASCVD score, and drug interactions. Add “. ASCVD" [an automated ASCVD risk calculator tool] in your note template to incorporate these data in your encounters for PLWH.

Patient resources: You all now have access to ".HIVSTATIN", a patient handout that you can automatically generate, which addresses Frequently Asked Questions (FAQ) regarding statin

prescribing for HIV patients. Please use this in your after-visit summary or secure message to patients

Data: We will send monthly updates with our current statin prescribing data. Our hope is to monitor and improve upon our numbers, especially those at highest risk

Best. Regards,

The QI project Team

Appendix B. Supplemental Table

Supplemental Table 1. Segmented regression analysis of statin prescription rates before and after the intervention. Baseline statin prescription rates and monthly prescribing trends are shown overall and by region (San Francisco and East Bay). Pre-intervention slope reflects the monthly change in prescribing before the intervention, immediate change represents the level change at the time of the intervention, and post-intervention slope reflects the monthly change after implementation. Estimates are reported as percent change per month with 95% confidence intervals.

Parameter	Overall [95% CI] p-value	San Francisco [95% CI] p-value	East Bay [95% CI] p-value
Baseline Statin Prescription (%)	44.08	43.68	45.02
Pre-Intervention Slope (%/month)	0.30 [0.25, 0.34]	0.23 [0.17, 0.29]	0.45 [0.39, 0.50]
Immediate Change (%/month)	0.34 [-0.78, 1.47] $p = 0.566$	-2.06 [-2.91, -1.22] $p < 0.001$	-0.83 [-1.53, -0.13] $p = 0.045$
Post-Intervention Slope (%/month)	1.24 [0.95, 1.53] $p < 0.001$	1.52 [1.32, 1.71] $p < 0.001$	1.64 [1.45, 1.83] $p < 0.001$
